# Ionophore Antibiotics Inhibit Type II Feline Coronavirus Proliferation In Vitro

**DOI:** 10.3390/v14081734

**Published:** 2022-08-06

**Authors:** Yoshikazu Tanaka, Eri Tanabe, Yuki Nonaka, Mitsuki Uemura, Tsuyoshi Tajima, Kazuhiko Ochiai

**Affiliations:** 1 Department of Veterinary Hygiene, Veterinary School, Nippon Veterinary & Life Science University, 1-7-1 Kyounan, Musashino 180-8602, Japan; 2 Research Center for Animal Life Science, Nippon Veterinary & Life Science University, 1-7-1 Kyounan, Musashino 180-8602, Japan; 3 Department of Veterinary Pharmacology, Veterinary School, Nippon Veterinary & Life Science University, 1-7-1 Kyounan, Musashino 180-8602, Japan

**Keywords:** feline coronavirus, ionophore antibiotics, feline infectious peritonitis, nigericin, valinomycin, salinomycin, cats

## Abstract

Feline coronaviruses (FCoVs) infect cats worldwide and cause severe systemic diseases, such as feline infectious peritonitis (FIP). FIP has a high mortality rate, and drugs approved by the Food and Drug Administration have been ineffective for the treatment of FIP. Investigating host factors and the functions required for FCoV replication is necessary to develop effective drugs for the treatment of FIP. FCoV utilizes an endosomal trafficking system for cellular entry after binding between the viral spike (S) protein and its receptor. The cellular enzymes that cleave the S protein of FCoV to release the viral genome into the cytosol require an acidic pH optimized in the endosomes by regulating cellular ion concentrations. Ionophore antibiotics are compounds that form complexes with alkali ions to alter the endosomal pH conditions. This study shows that ionophore antibiotics, including valinomycin, salinomycin, and nigericin, inhibit FCoV proliferation in vitro in a dose-dependent manner. These results suggest that ionophore antibiotics should be investigated further as potential broad-spectrum anti-FCoV agents.

## 1. Introduction

Coronaviruses (CoVs) are RNA viruses that belong to the order Nidovirales and family Coronaviridae [1,2]. CoV infection causes severe respiratory, enteric, renal, and neurological diseases in humans, cats, mice, swine, and birds. CoVs are classified into four genera according to their genotypic and serological characteristics [1,2]: Alphacoronavirus, Betacoronavirus, Gammacoronavirus, and Deltacoronavirus. Some severe secondary diseases caused by CoVs include transmissive gastroenteritis and feline infectious peritonitis (FIP) caused by Alphacoronavirus; severe acute respiratory syndrome (SARS), Middle East respiratory syndrome (MERS), coronavirus disease (COVID-19), and mouse hepatitis caused by Betacoronavirus; and avian infectious bronchitis caused by Gammacoronavirus [3].

Feline coronaviruses (FCoVs) are classified into two biotypes, feline enteric coronavirus (FECV) and feline infectious peritonitis virus (FIPV), based on their pathogenicity and clinical signs [4]. FECV induces mild or subclinical symptoms. Only a small population of FCoV-infected cats develop FIP, a severe systemic disease that results in fatality in cats [5]. Two antigenic serotypes are known: I and II. It is more difficult for type I FCoV to isolate viruses in cell culture than for type II FCoV [6]. Viral cell entry can occur in two ways. The first is cytosolic entry through early endosomes (type II FCoV), and the other is through late endosomes (type I FCoV) [7]. The cell receptor of type II FCoV is aminopeptidase N, which binds to the spike (S) protein and mediates the internalization of the virus into the cell [8,9]; however, the cell receptor of type I FCoV has not yet been identified. Nonetheless, both serotypes have been found to utilize dendritic-cell-specific intercellular adhesion molecule-grabbing nonintegrin to infect monocyte-derived dendritic cells [10]. Studies identifying the entry mechanisms of type II FCoV have been conducted using the 79-1146 strain. This strain is internalized through a novel clathrin- and caveolae-independent pathway, which depends on dynamin [11,12]. A low endosomal pH has been speculated to trigger conformational changes in the S protein of FCoV, releasing the virus from the endosome into the cytosol [13]. Cathepsin B, which is an endosome protease, plays an important role in cleaving the S protein of type II FCoV [13].

In recent studies, two types of antiviral agents have been developed to treat FIP (GS-441524 and GC376) [14,15]. However, passage of wildtype murine hepatitis virus in the presence of GS-5734 (remdesivir), which is a prodrug of GS-441524, resulted in two amino acid mutations in the nonstructural protein 12 (nsp12; polymerase) at the residues conserved across CoVs [16]. These mutations caused a 5.6-fold increase in resistance to GS-5734 based on 50% effective concentration values (EC50). SARS-CoV possessing the same amino acid mutations in nsp12 resulted in an identical in vitro resistance phenotype [16]. Therefore, the inhibitor might produce viral escape mutants. Additionally, higher dosages with GS-441524 or GC376 are required to transport the neural tissue through the blood–brain barrier [15,17]. Antivirals may target diverse viral and cellular processes [18]. Targeting host factors required for viral proliferation is advantageous for antiviral resistance because of minor mutations in the viral genome. In this study, we chose approved and available drugs that inhibit FCoV proliferation. It has been reported that ion chelators (ionophore antibiotics), including valinomycin (cyclodepsipeptide ionophore), salinomycin, and nigericin (polyether ionophore) exhibit antiviral activity in in vitro experiments [19]. These antibiotics strongly bind to cellular potassium ions and alter the ion concentrations and pH within the cell [20]. Originally, these ionophore antibiotics were known for their inhibitory activities against Gram-positive bacteria and coccidian protozoa in the veterinary field [19]. However, antiviral activities against diverse DNA and RNA viruses have also been reported, including human immunodeficiency virus-1 (HIV-1) [21], influenza virus [22], Zika virus [23], MERS-CoV [24,25], SARS-CoV [26], SARS-CoV-2 [27], mouse hepatitis virus [24], vesicular stomatitis virus [28], poliovirus [29], La Crosse virus [23], and hepatitis B virus [30].

Recently, Ke et al. [31] reported that salinomycin showed antiviral activity against FCoV at a concentration of 10 µM. However, little is known about its detailed inhibitory mechanisms. In this study, we show how ionophore antibiotics affect FCoV proliferation. We expect that ionophore antibiotics will be further investigated as potential broad-spectrum anti-FIP agents.

## 2. Materials and Methods

### 2.1. Cell Culture and Virus

*Felis catus* whole fetus-4 (Fcwf-4) cells were purchased from American Type Culture Collection (CRL-2787; Manassas, VA, USA) and maintained in Dulbecco’s modified Eagle’s medium (DMEM; Fujifilm WAKO Pure Chemical Corporation, Tokyo, Japan) supplemented with 5% fetal calf serum (FCS; JRH, Nissui, Tokyo, Japan). We propagated FIPV (79–1146 strain; a gift from Dr. Tsutomu Hodatsu, Kitasato University, Tokyo, Japan) in the Fcwf-4 cells.

### 2.2. Chemical Compounds

Chemical compounds were purchased as follows: valinomycin, salinomycin, nigericin, and niclosamide were purchased from MedChemExpress (South Brunswick, NJ, USA). All compounds except for nigericin were prepared as a 10 mM stock solution in dimethyl sulfoxide (DMSO), respectively. Nigericin was dissolved in ethanol to prepare a 10 mM stock solution. All compounds were stored at −20 °C.

### 2.3. Cells Treated with Compounds

The Fcwf-4 cells were seeded at a density of 2.5 × 10^5^ cells/well into 12-well plates. After an 18 h incubation period at 37 °C, the cells were treated with each compound at a twofold serial dilution (niclosamide; 0.8 μM to 0.0008 μM, valinomycin; 25 μM to 0.025 μM, salinomycin; 12.5 μM to 0.0125 μM, nigericin; 6.3 μM to 0.0063 μM) or each solvent (1% DMSO or 0.1% ethanol) in the medium for 1 h at 37 °C before infection with FIPV. We inoculated the Fcwf-4 cells with FIPV at a multiplicity of infection of one plaque-forming unit per cell to study the effect of each ionophore antibiotic against FIPV infection. The medium containing the virus was removed after 1 h of adsorption at 37 °C. The cells were rinsed three times with phosphate-buffered saline (PBS (-)) and incubated with or without each compound in fresh 5% FCS-DMEM for 20 h. The cells not treated with antibiotics were treated with a solvent (1% DMSO or 0.1% ethanol).

### 2.4. Cell Viability Assay

We assayed WST-8 to evaluate cytotoxicity using the Cell Counting Kit-8 (Dojin Chemical Inc., Toyama, Japan), according to the manufacturer’s instructions. Briefly, Fcwf-4 cells were seeded at 2 × 10^4^ cells/well into 96-well plates and incubated overnight at 37 °C before treatment with each compound. Each compound was added to the cells at a twofold serial dilution (100 μM to 0.1 μM). The cells were incubated in the presence of compounds for 24 h before addition of the WST-8 reagent. Absorbance was measured at 450 nm after incubation for 1 h with the WST-8 reagent. The results are plotted as the mean of three independent experiments.

### 2.5. Western Blot Analysis

The cell membranes were disrupted with cell lysis buffer (10 mM Tris-HCl, pH 7.8, 1 mM ethylenediaminetetraacetic acid (EDTA), 1% NP-40, 0.15 M NaCl), including cOmplete Mini (Roche Diagnostics, Tokyo, Japan) at 20 h after infection. The cell lysates were resolved by electrophoresis on 12.5% SuperSep gels (Fujifilm WAKO Pure Chemical Corporation) and Western blotting onto Immobilon-P membranes (Millipore, Tokyo, Japan). Non-specific protein binding was blocked with 5% non-fat dry milk for 1 h, and then the membranes were incubated with anti-feline coronavirus nucleocapsid (N) antibody (FIPV3-70; MyBioSource, San Diego, CA, USA) or anti-glyceraldehyde-3-phosphate dehydrogenase antibody (GAPDH, 6c5 clone; Calbiochem, Tokyo, Japan) for 1 h. Antigen signals were visualized by reacting proteins on the membranes with horseradish peroxidase-conjugated anti-mouse IgG antibody (Promega Corporation, Madison, WI, USA), followed by an enhanced chemiluminescence substrate (ImmunoStar LD; Fujifilm WAKO Pure Chemical Corporation, Tokyo, Japan), according to the manufacturer’s protocol. Signals were detected using the ImageQuant LAS 4000-mini Imaging System (GE Healthcare Life Sciences, Marlborough, MA, USA) and analyzed using Multi Gauge Version 3.0 software (GE Healthcare Life Sciences, MA, USA).

### 2.6. Reverse Transcription-Quantitative Polymerase Chain Reaction (RT-qPCR)

The number of viral genome copies in the Fcwf-4 cells infected with FCoV in each compound was determined using RT-qPCR [32]. Briefly, the medium was removed 20 h after infection, and RNA from the medium was prepared using Isogen-LS (Nippon Gene Co., Ltd., Toyama, Japan), according to the manufacturer’s protocol. Total RNA was reverse transcribed, and viral cDNAs were quantified using the THUNDERBIRD Probe One-step RT-qPCR kit (TOYOBO Co., Ltd., Tokyo, Japan) with specific primers for the gene encoding the FCoV nucleocapsid (GenBank: DQ010921.1; forward, 5′-TGGCCACACAGGGACAAC-3′; reverse, 5′-AGAACGACCACGTCTTTTGGAA-3′) and the TaqMan probe (FAM-TTCATCTCCCCAGTTGACG-BHQ-1). The sequences were amplified using a 7500 Sequence Detection System (Thermo Fisher Scientific, Tokyo, Japan).

### 2.7. Viral Titration

Viral titers were determined by end-point dilution assays. Fcwf-4 cells were seeded into 96-well plates at a density of 2 × 10^4^ cells/well and incubated overnight at 37 °C. The next day, the cells were inoculated with a 1/3 serial dilution of the virus sample. The cytopathic effect (CPE) was considered as a readout at 3 days post infection, and the 50% tissue culture infectious dose (TCID_50_) per mL was calculated according to the Reed and Muench method.

### 2.8. Calcium Imaging

The Fcwf-4 cells were seeded into a 3.5 cm glass bottom dish (Corning, Tokyo, Japan) 18 h before transfection. The cells were transfected with 2 µg pcDNA-LUCI-GEC01 (#113675; Addgene, Watertown, MA, USA), which was derived from pGCaMP6s, using 6 μL X-tremeGENE HP DNA Transfection Reagent (Roche Diagnostics, Tokyo, Japan), according to the manufacturer’s instructions. After the transfected cells had been incubated for 6 h, the medium was replaced with fresh 5% FCS-DMEM. The cells were incubated for 48 h, followed by treatment with phorbol 12-myristate 13-acetate (0.05 µg/mL, PMA; Sigma-Aldrich, Tokyo, Japan) with each compound or 0.3% DMSO for 60 min. The maximum concentrations of salinomycin, valinomycin, and nigericin without obvious cell toxicities (12.5 μM, 25 μM, and 6.3 μM, respectively) were added to the cells. Images were acquired using a laser scanning confocal microscope (LSM 710; Carl Zeiss, Tokyo, Japan). The image fields per dish were captured every 2 min for 60 min at an excitation of 460–490 nm and an emission of 500–550 nm. The images were analyzed using ImageJ-Fiji software version 1.53r (National Institutes of Health, Bethesda, MD, USA, http://imagej.nih.gov/ij/download.html, accessed on 28 February, 2022). The fluorescence intensity (F) was subtracted from the same frame in the first acquisition to remove the background signal. To calculate the ΔF/F0 index, F0 was considered the median baseline fluorescence before adding each compound, where ΔF = F − F0. Regions of interest per condition were compiled with a total of at least 50 green fluorescence-positive cells per condition.

### 2.9. pH Alteration Analysis with LysoGlow84

To examine alterations of pH in the lysosome by ionophore antibiotics, Fcwf-4 cells were seeded into a 12-well plate (FALCON, Tokyo, Japan) at a density of 2.5 × 10^5^ cells per well. Briefly, 10 μM of LysoGlow84 (AdipoGen Life Sciences, San Diego, CA, USA) with each antibiotic, i.e., salinomycin, valinomycin, and nigericin (12.5 µM, 25 μM, and 6.3 μM, respectively), or 0.3% DMSO at maximum concentrations without obvious cell toxicities was added to the cells. The cells were incubated for 2 h at 37 °C. The cells were detached using trypsin–EDTA solution and collected after adding 5% FCS and PBS. Finally, the fluorescence spectra were measured at 315 nm (excitation) and 360–560 nm (emission) using an FP-8050 spectrophotometer (Nihon Bunko, Tokyo, Japan).

### 2.10. Statistical Analysis

Data were analyzed using GraphPad Prism software (version 6.0, GraphPad Prism software, San Diego, CA, USA). Statistical significance of comparisons between one control group and multiple treatments was tested using one-way ANOVA with Dunnett’s multiple comparison test. Statistical significance was set at * *p* < 0.05 or ** *p* < 0.001.

## 3. Results

### 3.1. Cytotoxicity of Each Ionophore Antibiotic on the Fcwf-4 Cells

**Figure 1:**
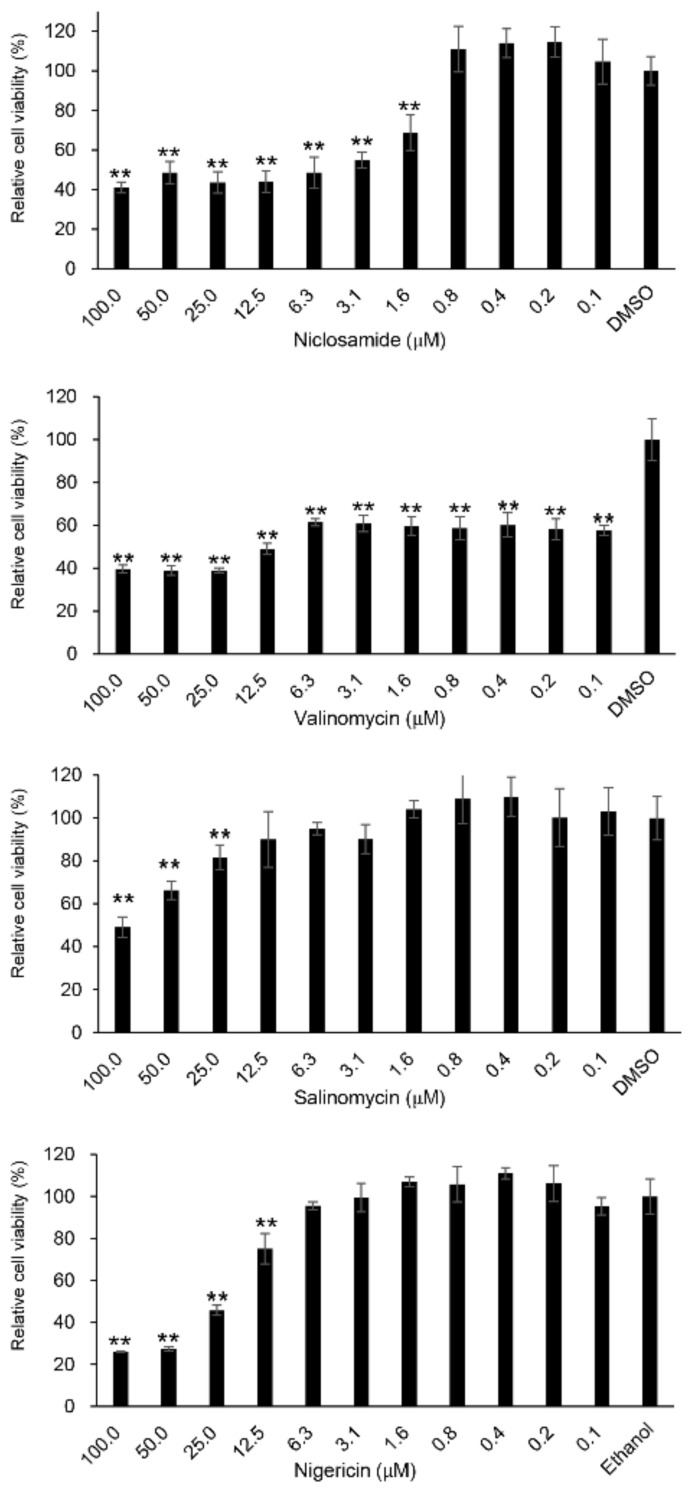
Cell viabilities with ionophore antibiotics. The *Felis catus* whole fetus-4 (Fcwf-4) cells were incubated with different types of ionophore antibiotics and niclosamide at various concentrations or a solvent (1% DMSO for niclosamide, valinomycin, and salinomycin, or 0.1% ethanol for nigericin) to examine cell toxicity. After 24 h treatment, cell viability was assessed by the WST-8 assay as described in the Materials and Methods section. Error bars indicate standard deviations (SD). Data were analyzed with one-way ANOVA with Dunnett’s multiple comparison test (** *p* < 0.001). Each graph column represents mean ± SD (N = 8).

First, WST-8 assays were performed to determine the viability of the Fcwf-4 cells treated with each compound using the method reported by Tanaka et al. [31]. Niclosamide, which blunts calcium oscillations, was used as the reference. Niclosamide has been identified as a potent agent against SARS-CoV-2, MERS-CoV, SARS-CoV, and FCoV in vitro [32,33,34,35]. All compounds were tested at concentrations ranging from 0.1 to 100 µM (Figure 1). Only valinomycin (0.4 µM) showed cytotoxicity in the WST-8 assays. However, there were no obvious alterations in the cell morphology at a concentration of 0.4 µM compared with the morphology of mock-treated cells (Figure S1).

### 3.2. Ionophore Antibiotics Inhibit FCoV Replication

**Figure 2:**
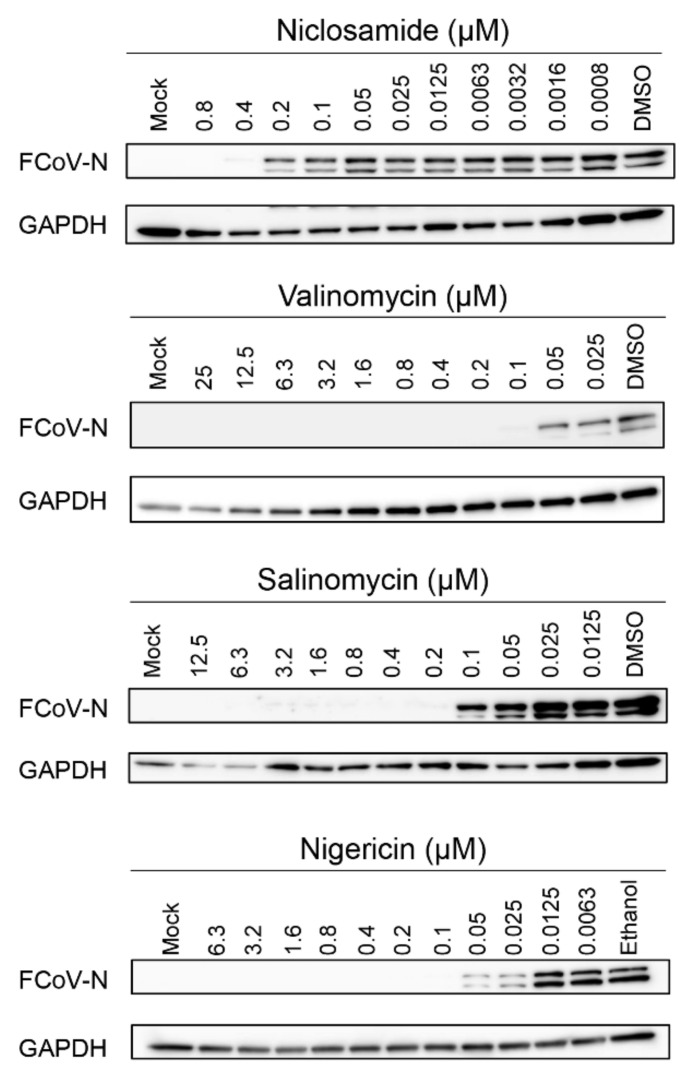
Ionophore antibiotics inhibit FCoV replication in a dose-dependent manner by Western blot analysis. The Fcwf-4 cells were incubated with ionophore antibiotics or an equivalent volume of each solvent after infection with FCoV in the presence of the antibiotics and niclosamide. At 20 h after infection, the cells were lysed with cell lysis buffer and assessed by Western blot analysis. FCoV, feline coronavirus; Fcwf-4, *Felis catus* whole fetus-4; GAPDH, glyceraldehyde-3-phosphate dehydrogenase; FCoV-N, feline coronavirus nucleocapsid.

**Figure 3:**
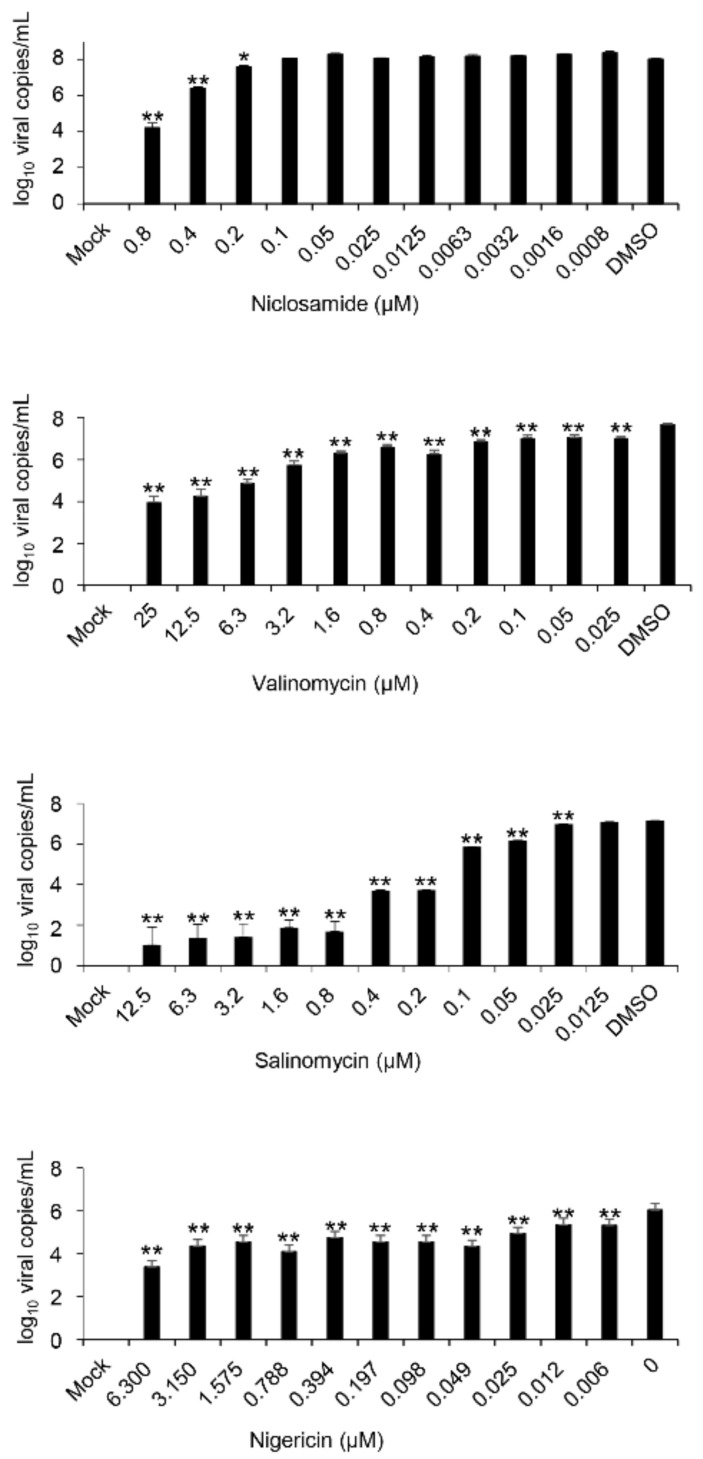
Ionophore antibiotics inhibit FCoV replication in a dose-dependent manner by RT-qPCR. Total RNAs from the supernatants of the infected cells were treated as described in the Materials and Methods section; they were extracted and analyzed by RT-qPCR. Each sample was assessed through triplicate measurements. Error bars indicate standard deviations. Data were analyzed with one-way ANOVA with Dunnett’s multiple comparison test (* *p* < 0.05; ** *p* < 0.001). FCoV, feline coronavirus; RT-qPCR, reverse transcription-quantitative polymerase chain reaction.

Next, we examined the inhibitory effects of ionophore antibiotics on FCoV replication by Western blot analysis (Figure 2). All the compounds suppressed the FCoV-N expression in a dose-dependent manner. Nigericin showed inhibitory effects at concentrations less than 0.05 µM based on the results of the Western blot analysis. Valinomycin and salinomycin completely suppressed the FCoV-N expression at 0.2 µM concentration, and complete suppression by niclosamide was at 0.4 µM. To quantify the viral copies in the supernatants of the infected cells with or without antibiotics, viral copies were quantified by RT-qPCR. All the antibiotics were used at concentrations without cell toxicity, as shown in Figure 2. Consequently, niclosamide and other ionophore antibiotics showed dose-dependent inhibitory effects against FCoV (Figure 3). These data were analyzed using RT-qPCR and Western blot analysis. Nigericin displayed the strongest inhibitory effects against FCoV replication (EC_50_ = 0.006189 μM) compared with salinomycin (EC_50_ = 0.03049 μM) and valinomycin (EC_50_ = 0.01580 μM). Remarkably, all ionophore antibiotics inhibited viral replication at lower concentrations than that by niclosamide (EC_50_ = 0.1761 μM). We also measured infectious viral titers based on the TCID_50_ assay. All infectious viruses in the supernatants of the cells treated with antibiotics at the concentrations of 0.8 µM niclosamide, 0.2 µM valinomycin, 0.4 µM salinomycin, and 0.05 µM nigericin, respectively, were not detected (Table 1). In conclusion, these results indicate that ionophore antibiotics have strong antiviral activities against FCoV type Ⅱ in Fcwf-4 cells.

### 3.3. Alteration of Calcium Ion Concentration in Cytoplasmic Compartments by Ionophore Antibiotics

**Figure 4:**
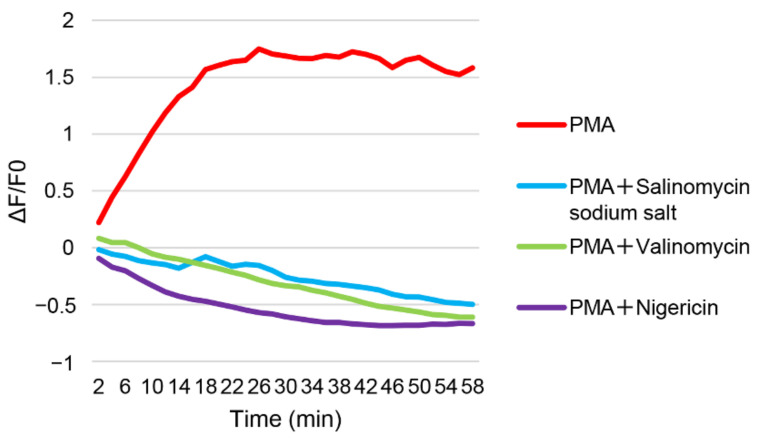
Ionophore antibiotics alter the Ca^2+^ concentration dynamics in cultured cells. The Fcwf-4 cells were transfected with pcDNA-LUCI-GEC01. The cells were incubated for 48 h, following treatment with PMA and salinomycin, valinomycin, or nigericin (12.5 μM, 25 μM, and 6.3 μM, respectively) at maximal concentrations that did not cause cell toxicity, or 0.3% DMSO for 60 min. Image acquisition was performed using a laser scanning confocal microscope. Images were analyzed using the ImageJ-Fiji software. Fcwf-4, *Felis catus* whole fetus-4; PMA, phorbol 12-myristate 13-acetate.

All antibiotics used in this study were monovalent cation ionophores, and were presumed to prevent the formation of proton gradients by vacuolar ATPase (v-ATPase) in cellular organelles [36,37]. Coronaviruses enter host cells via pH-dependent endocytosis, and the acidic environment of endolysosomes is regulated by v-ATPase, Na^+^/K^+^-ATPase, Niemann-Pick type C1, and two-pore channels [38,39,40,41]. These cellular factors also play important roles in endosomal trafficking by regulating Ca^2+^ concentration [38,39,40,41]. Ca^2+^ is important for viral entry and gene expression, and is involved in imbalanced Ca^2+^ homeostasis during viral infection [42,43,44]. Therefore, we examined whether ionophore antibiotics affected the calcium concentration in the cells. Many studies have been conducted in neurology using pcDNA-LUCI-GEC01, which is derived from pGCaMP6s, to monitor Ca^2+^ concentration dynamics in cultured cells [45]. PMA is a well-known stimulator of calcium signaling in cells. We also examined the effect of PMA on the calcium signaling stimulation in the Fcwf-4 cells. PMA evidently enhanced the signals, but all antibiotics suppressed the Ca^2+^ concentration in 30 min (Figure 4). Salinomycin, nigericin, and valinomycin prevented the increase in the Ca^2+^ concentration.

### 3.4. Ionophore Antibiotics Alter pH in the Endosomes

**Figure 5:**
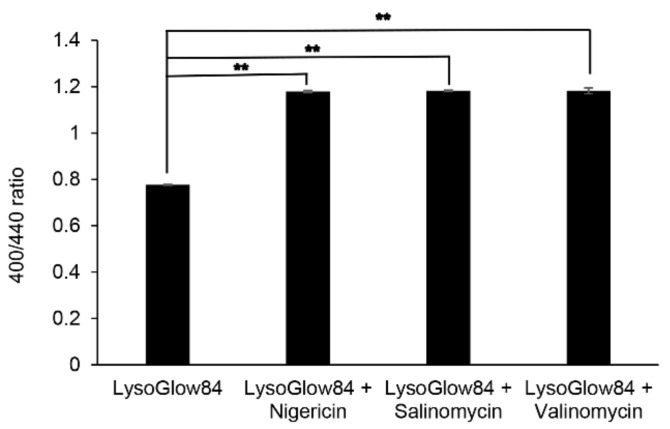
Ionophore antibiotics affect the pH of endosomes. LysoGlow84 (10 μM) with each antibiotic of salinomycin, valinomycin, and nigericin (12.5 μM, 25 μM, and 6.3 μM, respectively) at maximal concentrations without cell toxicities was added to the *Felis catus* whole fetus-4 cells. Fluorescence spectra were measured at 315 nm (excitation) and 360–560 nm (emission) using a spectrophotometer. The alkaline range from pH 8 to 13 showed fluorescence intensity at 400 nm emission, and those of the acidic range from pH 3 to 6 at 440 nm emission and at 315 nm excitation. Increasing the ratio of 400/440 indicates shifting to alkaline conditions. Error bars indicate standard deviations. Data were analyzed using one-way ANOVA with Dunnett’s multiple comparison test (** *p* < 0.001).

Acidic conditions allow the transmembrane protease serine 2 and cathepsins B and L to cleave the S protein of coronavirus; subsequently, the S protein fuses with the host cell membranes to enter the cytosol [46]. Therefore, we examined whether ionophore antibiotics affect the pH conditions in lysosomes. It is well known that the fluorescence dye, LysoGlow84, is localized in the endosomes [47]. It has been reported that the alkaline range from pH 8 to 13 showed fluorescence intensity at 400 nm emission, and those of the acidic range from pH 3 to 6 at 440 nm emission and 315 nm excitation. Therefore, increasing the ratio of 400/440 indicates a shift to alkaline conditions [47]. Consequently, all compounds allowed pH alterations to shift from acidic to alkaline conditions in the endosomes using the dye (Figure 5). These results indicate that the ionophore antibiotics used in this study may suppress FCoV replication by altering Ca^2+^ concentrations and deacidification.

## 4. Discussion

The results of this study show that all the ionophore antibiotics used in this study inhibited FCoV replication in a dose-dependent manner, and the effects might be due to alterations in the Ca^2+^ concentrations and pH in endosomes. Ionophore antibiotics facilitate the movement of specific ions across cellular membranes, and can be divided into two groups. One is electrogenic, and the other is an electroneutral group (polyether) [22]. Electrogenic ionophores, including valinomycin, transfer net charge across the membrane. However, polyether ionophores, such as nigericin and salinomycin, facilitate electrically neutral cation exchange diffusion [20]. Polyether ionophores possess inhibitory activities against cancer, coccidian protozoa, Gram-positive bacteria, and drug-resistant strains [20,48,49]. In addition, these compounds have antiviral activities against HIV-1 [20], influenza virus [22], Zika virus [50], and SARS-CoV-2 [19]. Ion channel inhibitors, including salinomycin and monensin, have been screened as anti-MERS-CoV and anti-SARS-CoV inhibitors from 290 drugs. These compounds can inhibit the cytopathic effects of MERS-CoV [25]. However, the antiviral mechanisms of ionophores remain unclear. Therefore, we first examined whether valinomycin, salinomycin, and nigericin suppressed FCoV proliferation. Next, we investigated whether ionophore antibiotics affected the Ca^2+^ concentration dynamics and endosomal pH alterations in cultured cells. Consequently, all antibiotics inhibited FCoV replication and altered the Ca^2+^ concentration and endosomal pH.

Viral entry into cells is the most critical step of the viral life cycle. FCoV binds to its cellular receptor, and the virus requires access to the cytoplasm to perform viral replication. The complex between the viral receptor and the viral S protein is endocytosed, following the cleavage of the S protein by cathepsin B to fuse with the endosomal membrane [51]. The fusion peptide of the S protein is located in the S2 domain. There are either one or two proteolytic activities of the S protein: Type I FCoV has two specific activation sites; S1/S2 cleaved by furin-like protease, and S2′ cleaved by cathepsin B. Type II FCoV only has the S2′ site, cleaved by cathepsin B [51]. Two essential events are required to induce fusion: cleavage of the S protein, and a drop in the endosome pH [14,52]. This pH drop is necessary for virus–membrane fusion, proteolytic activation, and conformational changes in the S2′ domain [14,53,54]. In addition, an ionic factor, i.e., Ca^2+^, enhances viral membrane fusion. Ca^2+^ directly interacts with the fusion peptide in the S proteins of SARS-CoV, SARS-CoV-2, and MERS-CoV, and the GP protein of the Ebola virus [44,55]. In this study, ionophore antibiotics changed the pH of the endosomes and decreased Ca^2+^ concentrations. Therefore, it may be presumed that these antibiotics inhibit viral entry into the cytosol and replication. Ke et al. [31] have reported that during artificial intelligence screening for drugs, five drugs, including salinomycin, inhibited FCoV proliferation in Fcwf-4 cells. In particular, salinomycin exhibited its antiviral activities at a concentration of 10 μM. However, the results of the TCID_50_ assay indicate that salinomycin inhibits FCoV proliferation at a concentration one-tenth or less than that reported by Ke et al. [31]. Furthermore, Yang et al. [56] have reported that valinomycin, salinomycin, and niclosamide exhibited inhibitory activities against FCoV in vitro. However, their compounds also required higher concentrations to inhibit the virus compared with those used in our study. It is likely that these differences were derived from different experimental methods and conditions. It is well known that the RT-qPCR assay is highly sensitive in detecting viral genes. In this study, there were discrepancies in the results between Western blot and RT-qPCR analysis when using the same concentration of compounds (Figure 2 and Figure 3). One explanation for the discrepancies may be that RT-qPCR can detect nucleotides, including small disrupted cDNA fragments of the viral gene, even if mature viral particles are not constructed. Comparison of data obtained with the Western blot analysis and TCID_50_ assays revealed no significant differences. Therefore, these findings, as well as our study findings, indicate that ionophore antibiotics may exhibit antiviral activities at low concentrations in in vitro studies.

Treatments with ionophore antibiotics are generally not used in medical and veterinary applications because they are toxic to some animals. Horses and other equids are extremely sensitive to ionophore action, and cases of poisoning in cattle, sheep, turkeys, cats, dogs, and rabbits have also been described [57,58,59,60,61,62,63]. However, ionophore antibiotics are generally safe and effective at a therapeutic level in animals. Ionophores such as monensin, lasalocid, and salinomycin are used in animal feed to prevent coccidiosis, and increase feed efficiency in poultry and cattle [63,64,65]. The salinomycin used in this study showed inhibitory effects against FCoV replication at low concentrations. Borlle et al. [66] reported that salinomycin decreases feline sarcoma and carcinoma cell viability. In their report, they pointed out that 5 μM of salinomycin can achieve dose-dependent effects in vivo. Furthermore, Linde-Sipman et al. [62] reported that provocation testing did not result in clinical signs in the two cats tested, suggesting adverse events may not result in greater morbidity and mortality than that seen with cytotoxic chemotherapeutics. The concentration of salinomycin used in our study to inhibit viral proliferation is less than the chemotherapeutic dose. However, we must consider potential tolerability and toxicity of salinomycin in cats. Therefore, if ionophore antibiotics can be applied for the treatment of FIP without side effects, they may provide the possibility of effective drugs for cats with FIP. In addition, the development of medicinal chemistry for structural modification to decrease toxicity is necessary in clinical trials. Moreover, ionophore antibiotics should be further investigated as potential broad-spectrum anti-FCoV agents.

## Figures and Tables

**Table 1:** Antiviral activities of ionophore antibiotics in Fcwf-4 cells based on TCID_50_ assay.

Antibiotics or Solvents	Viral Titer (TCID_50_/mL)	SD
Niclosamide (0.8 µM)	N.D.	-
Valinomycin (25 µM)	N.D.	-
Salinomycin (12.5 µM)	N.D.	-
Nigericin (6.3 µM)	N.D.	-
DMSO (0.3%)	5.5 × 10^7^	1.6 × 10^7^
Ethanol (0.1%)	4.4 × 10^7^	1.9 × 10^7^

N.D., not detected; SD, standard deviation. Viral titers were assessed through triplicate measurements.

## Data Availability

The authors confirm that all data analyzed and presented in this study are available on request from the corresponding author.

## Supplementary Materials

The following supporting information can be downloaded at: https://www.mdpi.com/article/10.3390/v14081734/s1. Figure S1: Cytotoxicity of each ionophore antibiotic on the Fcwf-4 cells.
