# Supplementary material for: Ionophore Antibiotics Inhibit Type II Feline Coronavirus Proliferation In Vitro
Source: Viruses. 2022 Aug 6;14(8):1734. doi: 10.3390/v14081734 (PMC9415497; doi:10.3390/v14081734)
Supplement: Supplementary file 1 [file viruses-14-01734-s001.zip › viruses-1780290-supplementary.pdf]

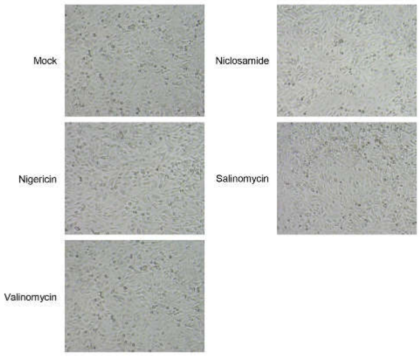

Figure S1. Cytotoxicity of each ionophore antibiotic on the Fcwf-4 cells. Each ionophore antibiotic does not obviously affect cell morphology. The Fcwf-4 cells were incubated with niclosamide (0.8 μM), nigericin (6.3 μM), salinomycin (12.5 μM), and valinomycin (25 μM) for 20 h at 37 °C. Original magnification, × 100. Fcwf, *Felis catus* whole fetus-4
